# Effects of three products from Antarctic krill on the nitrogen balance, growth, and antioxidation status of rats

**DOI:** 10.1002/fsn3.1140

**Published:** 2019-07-16

**Authors:** Xiaoming Ma, Chuyi Liu, Changwei Wang, Xiaoying Ma, Shuai Che, Xiaomei Feng, Bafang Li, Yuankun Dai

**Affiliations:** ^1^ College of Food Science and Engineering Ocean University of China Qingdao China; ^2^ School of Medicine and Pharmacy Ocean University of China Qingdao China; ^3^ Marine Biomedical Research Institute of Qingdao Qingdao China; ^4^ Apeloa Pharmaceutical Co. Ltd. Dongyang China; ^5^ College of Marine Life Sciences Ocean University of China Qingdao China

**Keywords:** antarctic krill, antioxidant activity, growth, krill powder, krill protein complex, nitrogen balance

## Abstract

A few studies conducted over the past few decades have demonstrated the health benefits of a diet rich in marine products, but limited studies have investigated the effects of different krill products on the nitrogen balance and their potential health benefits. In our study, after a 14‐day acclimation period, 50 female Sprague–Dawley rats were randomly assigned to five groups, each of which was fed a different diet, for 28 days. We then evaluated the effect of krill protein complex (KPC), krill powder, and defatted krill powder on the nitrogen balance, growth, and antioxidant activity through analyses of MDA, CAT, GSH‐Px, and T‐SOD. An in vivo analysis suggested that the nitrogen retention rate, protein digestibility, and bioutilization of krill products were equal to those of casein. Moreover, the KPC diet resulted in the highest nitrogen intake and retention among the groups, and the biological value and net protein utilization obtained with KPC were higher than those obtained with defatted krill powder, which was consistent with the weight gains observed for these two groups. The hematological test also showed that KPC contributed to the production of functional proteins in the body. The antioxidant activity analysis indicated that higher GSH‐Px and T‐SOD activities were obtained with krill products and KPC, respectively, compared with casein. The results from this study suggested that krill proteins could promote growth and improve the antioxidant status of an organism. Although further studies on the safety of krill products for human consumption are needed, this work provides insights into the use of krill proteins as a potential substitute for other proteins and restructured foods.

## 1 INTRODUCTION

Protein‐calorie malnutrition remains a public health problem in many developing nations throughout the world (Nisar et al., 2016). Proteins are important nutrients because they participate in growth and development, and their amino acids are used to produce enzymes, hormones, neuropeptides, immunoglobulins, receptors, and transporters. Additionally, proteins are important because they contribute to organism function (Loenneke, Loprinzi, Murphy, & Phillips, 2016; Sahni, Mangano, Hannan, Kiel, & McLean, 2015) and the maintenance of general health (Gregorio et al., 2014).

Many studies have shown that the long‐term consumption of red meat is associated with all‐cause mortality (Larsson & Orsini, 2014; Pan et al., 2012; Sinha, Cross, Graubard, Leitzmann, & Schatzkin, 2009), cardiovascular diseases (Micha, Michas, & Mozaffarian, 2012; Micha, Wallace, & Mozaffarian, 2010), type 2 diabetes (Aune, Ursin, & Veierod, 2009; Micha et al., 2010; Pan et al., 2011), certain types of adenocarcinomas (Aune et al., 2013; Cross et al., 2010; Cross & Sinha, 2004), age‐dependent macular degeneration (Chong et al., 2009; Ersoy et al., 2014), and possibly other inflammatory processes (Benito‐Garcia, Feskanich, Hu, Mandl, & Karlson, 2007; Choi, 2004; Oliver & Silman, 2006), which has resulted in a worldwide trend to reduce red meat from the human diet. In corroboration with the abovementioned facts, the health benefits of a diet rich in marine products have been demonstrated in a few studies conducted over the past few decades. In addition to studies that have extensively investigated the benefits of marine polyunsaturated fatty acids, recent scientific research has focused on marine proteins as potentially important compounds for human health (Aksnes, 2005; Khora, 2013; Ngo, Vo, Ngo, Wijesekara, & Kim, 2012).

Antarctic krill (*Euphausia superba*) is a zooplankton crustacean rich in protein and lipids (Aksnes, 2005; Tou, Jaczynski, & Chen, 2007), and its total biomass is 117–379 million metric tons (Atkinson, Siegel, Pakhomov, Jessopp, & Loeb, 2009). Antarctic krill is the largest animal protein resource in the world (Wang, Xue, Wang, & Yang, 2011), and its protein content is estimated to be in the range of 60% to 65% dry weight (Nicol, Forster, & Spence, 2000). Similar to other animal foods, the proteins derived from krill are considered complete proteins due to the presence of all nine essential amino acids required by adults (Chen, Tou, & Jaczynski, 2009). Additionally, the biological value of krill proteins is higher than those of meat and milk proteins (Suzuki & Shibata, 1990). Based on its nutrient composition, krill proteins might be a potential substitute for other proteins and restructured value‐added food products for the human diet. Although krill has the benefits of having high levels of n‐3 polyunsaturated fatty acids (PUFAs) and high‐quality proteins, only approximately 12% of total krill production is consumed by humans (Ichii, Imai, & Irimura, 2000). Due to its abundance and underutilization, krill is a relatively untapped potential protein source for humans.

A constant supply of high‐quality proteins is needed for the maintenance of growth and other physiological functions (Boye, Wijesinha‐Bettoni, & Burlingame, 2012). Several studies have focused on the quality of krill proteins. Iwantani, Obtake, and Tamura (1977) and Gigliotti, Jaczynski, and Tou (2008) assessed the nutritional value of krill proteins through animal experiments, and based on their studies, krill appears to be a promising protein source for human consumption. Due to the limited number of studies on the role of different krill products on the nitrogen balance and oxidative status of the body, our study aimed to evaluate the effects of three types of krill products, namely krill protein complex (KPC), krill powder, and defatted krill powder, on the nitrogen balance. We also examined the scientific evidence regarding the health benefits of krill, including growth acceleration and antioxidant activity.

## 2 MATERIALS AND METHODS

### 2.1 KPC isolation

Frozen Antarctic krill (*Euphausia superba*), krill powder, and defatted krill powder were obtained from China National Fisheries Corp., Beijing, China, and were stored in a laboratory at −20℃ until use.

Proteins were isolated from frozen whole krill using an isoelectric point solubilization/precipitation method according to Chen and Jaczynski (2007). The resulting sediment was collected as KPC, freeze‐dried, and stored at −20℃.

### 2.2 Proximate analysis

The proximate compositions, including crude protein, lipid, moisture, ash, and mineral content, of whole Antarctic krill, freeze‐dried KPC, krill powder, and defatted krill powder were determined.

The amount of crude protein was measured using the Kjeldahl method according to the Chinese standard GB/T5009.5‐2016. The lipid content was determined through the Soxhlet extraction method using a Soxhlet apparatus according to the Chinese standard GB/T5009.6‐2016. The moisture was measured by the direct drying method according to the Chinese standard GB/T5009.3‐2016. The ash content was determined by the burning/weighing method according to the Chinese standard GB/T5009.4‐2016. The mineral contents (calcium, phosphorus, iron, zinc, copper, magnesium, and manganese) were measured by flame atomic absorption spectrophotometry (Shimadzu AA6880, Japan) according to the Chinese standards GB/T5009.92‐2016, GB/T5009.87‐2016, GB/T5009.90‐2016, GB/T5009.14‐2017, GB/T5009.13‐2017, GB/T5009.241‐2017, and GB/T5009.242‐2017, respectively.

### 2.3 Animal feeding study

All animal procedures were approved by the Animal Care and Use Committee at Ocean University of China. Immature (aged 28 days), female Sprague‐Dawley rats were individually housed in a metabolic cage; throughout the experiment, their food intake was measured, and their urine and feces were collected. The rats were housed in rooms maintained at 25°C with a 12‐hr light/dark cycle. During a 14‐day acclimation period, the animals were given ad libitum access to ddH_2_O and designed diets that meet all the nutrient requirements for growing rats.

Following the acclimation period, the rats (*n* = 50) were randomly assigned to one of five groups, and each group was fed one of the following five diets ad libitum: (1) 10% crude protein supplied as casein for 4 weeks (*n* = 10); (2) 10% crude protein supplied as casein for 1 week followed by a protein‐free diet for 3 weeks (*n* = 10); (3) 10% crude protein supplied as KPC for 4 weeks (*n* = 10); (4) 10% crude protein supplied as krill powder for 4 weeks (*n* = 10); and (5) 10% crude protein supplied as defatted krill powder for 4 weeks (*n* = 10). The amount of protein replaced by KPC, casein, krill powder, or defatted krill powder in the designed diets (10% of the total protein in the diets) was corrected for protein and lipids to ensure that the diets were isocaloric. The calcium (Nalsen et al.) and phosphorus (P) contents of the diets were also matched (Table 1). The assigned diets and ddH_2_O were analyzed, and fresh feed was given every 2 days. The body weight and length of all the animals were measured once a week.

**Table 1:** Designed diet composition

Ingredients (g/100 g diet)	10% Casein	Protein free	10% KPC	10% krill powder	10% defatted krill powder
Casein	10	0	0	0	0
DL‐methionine	0.15	0	0	0	0
KPC	0	0	12.5	0	0
Krill powder	0	0	0	14.5	0
Defatted krill powder	0	0	0	0	12.5
Sucrose	53	60	51.3	54	53
Corn starch	21	22	21.9	20	20.4
Corn oil	5.2	6	3.5	3.9	5
Cellulose	5.05	6.5	5	2.4	3.6
Vitamin mix^a^	1	1	1	1	1
Ethoxyquin	0.001	0.001	0.001	0.001	0.001
Mineral mix^a^	1.5	1.5	1.5	1.5	1.5
Calcium phosphate	2.5	2.5	2.5	0	0
Sodium dihydrogen phosphate	0	0	0.2	3.2	3.5
Calcium carbonate	0.6	0.5	0.6	0	0
Gross energy (Kcal/g)	3.7	3.6	3.7	3.6	3.6

Abbreviation: KPC, krill protein complex.

a Based on the AIN‐93G vitamin and mineral mixes (Reeves, Rossow, & Lindlauf, 1993).

Urine and fecal samples were collected and analyzed once a week. The urine samples collected each week were mixed, centrifuged at 5788 *g* for 10 min, aliquoted into fresh tubes, and stored at −20℃ until their use in nitrogen content assays. The fecal samples were blended, dried for 48 hr, and then stored at room temperature until their use in nitrogen content assays.

### 2.4 Nitrogen balance measurements

The nitrogen content was measured using the Kjeldahl method, and nitrogen balance parameters were determined. The protein quality was evaluated according to the method described by Pellet and Young (1980).

### 2.5 Organ coefficient

The spleen, liver, kidney, and heart of the rats were excised and weighed. The relative weight of each organ was calculated based on the final body weight measured on the day of organ collection. The organ coefficients were calculated as follows:

Organ coefficient (g/100 g) = organ weight/rat body weight × 100.

### 2.6 Hematological test

At the end of the experimental period, the rats were fasted for 12 hr and then anesthetized with ether. Blood samples were collected from the abdominal aorta into vessels containing a blood anticoagulant agent for immediate routine blood tests. The samples were also collected into centrifuge tubes to separate the serum, which was achieved by centrifugation at 4,000 rpm/min for 10 min and was stored at −80°C for future analyses.

The hemoglobin (Hb) level was measured with an Hb test solution (Nanjing Jiancheng Bioengineering Institute, Nanjing, China). The serum albumin level was determined with an albumin assay kit, and the level of blood urea nitrogen (BUN) was tested using a urea assay kit (Nanjing Jiancheng Bioengineering Institute, Nanjing, China). The serum transferrin levels were measured through a double‐antibody sandwich enzyme‐linked immunosorbent assay (Elabscience Biotechnology Co., Ltd.).

### 2.7 Antioxidant activity

Serum malondialdehyde (Xia, Bamdad, Ganzle, & Chen, 2012), catalase (CAT), total superoxide dismutase (T‐SOD), and glutathione peroxidase (GSH‐Px) were determined using commercial kits (Jiancheng Biotech, Nanjing, China). All other chemicals used in this study were of analytical grade.

### 2.8 Statistical analyses

A completely randomized design was used in this study. The differences between casein and different krill products were assessed using a *t* test, and the differences among the five diets were evaluated by one‐way ANOVA. A repeated‐measures mixed model analysis was used to analyze the body weight gain and length increment during the feeding study. Both the covariate and repeated‐measures calculations were performed using SPSS17.0 (SPSS Inc., Chicago, IL, USA). All differences with *p* < .05 were considered significant.

## 3 RESULTS AND DISCUSSION

### 3.1 Proximate analysis

The composition of whole Antarctic krill on a wet basis was 80.72% moisture, 14.57% crude protein, 1.68% total lipid, and 2.82% total ash, consistent with the study conducted by Grantham (1977). The proximate compositions of the KPC, krill powder, and defatted krill powder are shown in Table 2. The contents of crude protein in the KPC and defatted krill powder were higher than that in krill powder, whereas the content of total lipid in the KPC was higher than those in the other two krill products. The crude protein values obtained for the krill powder and KPC in this study were lower than those obtained by Gigliotti et al. (2008), who reported values of 76.5% and 77.75%, respectively. However, our values were consistent with those obtained in previous studies, which reported a proximate composition of krill on a dry weight basis of 45%–80% crude protein, 7%–30% total lipid, and 10%–20% total ash (Grantham, 1977; Savage & Foulds, 1987; Sidhu, Montgomery, Holloway, Johnson, & Walker, 1970). These findings showed that these three krill products are rich in minerals, such as calcium, zinc, iron, and magnesium.

**Table 2:** The proximate composition of krill protein complex, krill powder, and defatted krill powder

Composition (/100 g dry weight)	KPC	Krill powder	Defatted krill powder
Moisture	2.47	5.71	5.11
Crude protein	74.75	62.24	74.02
Total lipid	14.30	12.51	0.61
Ash	1.84	10.24	12.90
Others	6.64	9.30	7.36
Minerals			
Ca (mg/g)	1.25	20.66	13.67
P (mg/g)	3.8	15	12
Fe (mg/kg)	16	185	112
Zn (mg/kg)	89	132	106
Cu (mg/kg)	107	78	83
Mg (mg/kg)	355	6,406	5,179
Mn (mg/kg)	0.44	15	3.4
Ca:P	1:3.04	1.38:1	1.14:1

Abbreviation: KPC, krill protein complex.

### 3.2 Nitrogen balance assessment

The nitrogen intake, urine nitrogen, fecal nitrogen, nitrogen retention, digestibility (D), biological value (BV), net protein utilization (NPU), and protein efficiency ratio (PER) are shown in Table 3. The nitrogen intake and nitrogen retention values of the KPC‐fed group were higher compared with those of the other three groups (*p* < .05). The defatted krill powder‐fed group and the casein‐ and KPC‐fed groups exhibited the highest (*p* < .05) and lowest (*p* < .05) urinary nitrogen excretion values, respectively. No significant differences in fecal nitrogen excretion and nitrogen retention, which is expressed as a percentage of intake, were found among the groups.

**Table 3:** Nitrogen balance in different groups

	Casein	KPC	Krill powder	Defatted krill powder
Nitrogen intake (g/day)	0.22 ± 0.01	0.30 ± 0.07^a^	0.20 ± 0.01^bc^	0.25 ± 0.02^b^
Ureic nitrogen excretion (g/day)	0.004 ± 0.001	0.004 ± 0.003	0.007 ± 0.003^abc^	0.013 ± 0.002^ab^
Fecal nitrogen excretion (g/day)	0.01 ± 0.01	0.02 ± 0.005	0.01 ± 0.01	0.02 ± 0.02
Total nitrogen excretion (g/day)	0.02 ± 0.01	0.02 ± 0.005	0.02 ± 0.01	0.03 ± 0.02^a^
Nitrogen retention (g/day)	0.20 ± 0.02	0.27 ± 0.06^a^	0.18 ± 0.01^bc^	0.22 ± 0.02^b^
Nitrogen retention rate (% nitrogen intake)	92.03 ± 6.29	92.16 ± 0.85	90.62 ± 6.17	87.97 ± 6.33
Digestibility (%)	93.40 ± 4.06	93.70 ± 3.74	93.83 ± 4.10	92.36 ± 3.54
Biological value	0.92 ± 0.49	0.93 ± 0.51	0.90 ± 0.07	0.86 ± 0.06^ab^
Net protein utilization (%)	85.63 ± 9.07	87.20 ± 8.06	82.58 ± 12.08	77.02 ± 11.25^b^
Protein efficiency ratio (g body wt/g protein)	1.32 ± 0.32	1.52 ± 0.38	1.56 ± 0.49^ac^	1.34 ± 0.31

Note

Values are presented as mean ± *SD*; significant differences are indicated with different letters in the same row (*p* < .05). ^a^ *p* < .05 compared to casein group, ^b^ *p* < .05 compared to krill protein complex group, and ^c^ *p* < .05 compared to defatted krill powder group.

In addition, no significant differences in the D, NPU, and PER were found between krill product‐fed rats and the casein‐fed rats. The effectiveness with which a nitrogen balance can be achieved with a given amount of absorbed dietary nitrogen is defined in terms of BV. The NPU, which is the product of BV and NPU, is the amount of protein in food that is actually utilized by the body (Iwantani et al., 1977). Therefore, the higher BV (*p* = .009) and NPU (*p* = .032) values obtained for the KPC‐fed group compared with the defatted krill powder‐fed group indicate that the defatted krill powder‐fed rats showed less nitrogen retention and poorer bioavailability than the KPC‐fed rats. In the current study, no difference in PER was found among the rats fed different protein sources.

Adequate protein intake is necessary for a positive nitrogen balance and the synthesis of the structural components of muscles and other body tissues, as well as enzymes, hormones, and hemoglobin. Our results indicated that all the groups had a positive nitrogen balance, and the KPC group exhibited the highest nitrogen retention, BV, and NPU. The study conducted by Iwantani et al. (1977) showed that the protein of precooked (freeze‐dried and defatted) krill results in almost equal weight gain, PER, and net protein ratio (NPR) values as that of casein, although the values of these measurements were obviously lower than those obtained with whole egg protein. Kobatake, Ishiguro, Hirahara, Innami, and Nishide (1980) determined the nutritive value of protein in krill meat after removal of the exoskeleton and found that it remained inferior to that of egg protein. The nutritional values of krill protein concentrates were also determined. Savage and Foulds (1987) used laboratory rats and found that the PER obtained with krill protein was lower than those obtained with egg albumin and white fish meal. In contrast, Suzuki and Shibata (1990) reported that the BV of krill protein concentrate was higher than those of other meat proteins and milk protein (casein) but lower than that of whole egg protein. In contrast, Gigliotti et al. (2008) indicated that the BV and NPU of rats fed krill protein concentrate were lower than those of casein‐fed rats, but the weight gain, PER, and NPR obtained with krill protein were similar to those obtained with casein. In a human trial, Tamura (1980) fed boiled krill or whole egg to adult men for 21 days, obtained NPU values of 55% and 61%, respectively, and found no differences in their digestibility. Based on the above‐described evidence, krill appears to be a good source of high‐quality protein, and the large biomass and high‐quality protein offered by krill provide a safe and economical replacement for commercially available protein sources.

### 3.3 Effect on growth

#### 3.3.1 Changes in body length and weight

**Figure 1:**
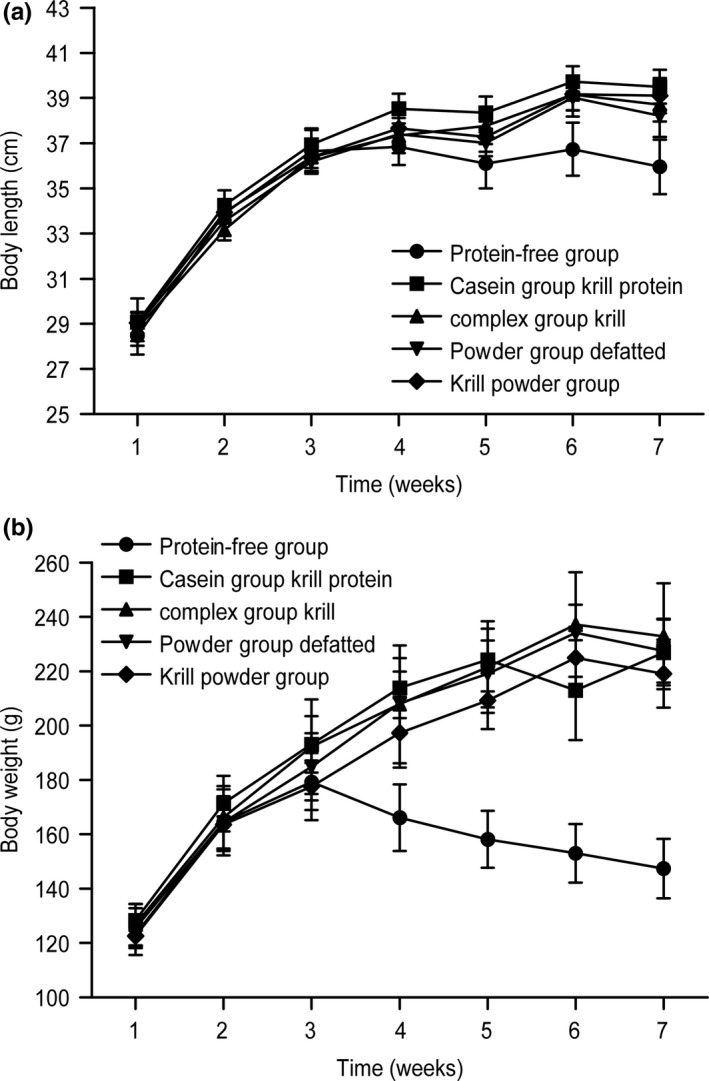
(a) Body lengths and (b) body weights of rats fed with different designed diets. Time includes the initial, acclimation feeding, and terminal periods. Values are presented as the mean ± *SD*

The body lengths and weights of growing rats, including those at baseline and at the acclimation, feeding and terminal periods, are shown in Figure 1. Throughout the study period, the body lengths and body weights of the groups fed casein and the three krill products increased substantially and significantly compared with those of the group that was not fed any protein (*p* < .05), and these effects were accompanied by a positive nitrogen balance in all the groups. A repeated‐measures analysis showed no significant difference among the groups fed the different protein sources. However, the KPC‐fed rats exhibited a higher weight than the defatted krill powder‐fed rats (*p* < .05), consistent with the above‐described data on nitrogen balance and protein quality. In addition, the body length of the groups fed krill products grew slightly slower than that of the casein‐fed group; however, no significant difference in body length was found between the krill product‐fed groups and the casein‐fed group at the end of the feeding period. These results suggest that casein is better digested by the body due to its bioavailability. When the proteins in krill extract or other krill products are introduced, the body needs time to adjust to digesting this new protein source. In addition, the height growth potential and overall shape of an individual are likely to be achieved through the regulation of bone growth (Millward, 1995), and more time might be needed before krill proteins can contribute to the regulation of bone growth. Furthermore, the body length is genetically determined, and each individual follows a growth curve canalized in terms of both extent and time course under favorable conditions (Tanner & Faulkner, 1986); of course, this growth curve is subjected to optimal fetal programming, which influences the postnatal height growth. Clearly, favorable conditions include adequate nutrition, in which dietary proteins play a key role (Millward, 1995). Sidhu et al. (1970) and Gigliotti et al. (2008) also reported that the KPC‐fed rats showed a similar weight gain and comparable protein efficiency ratios as the casein‐fed rats.

#### 3.3.2 Organ coefficients of rats and hematological test

**Figure 2:**
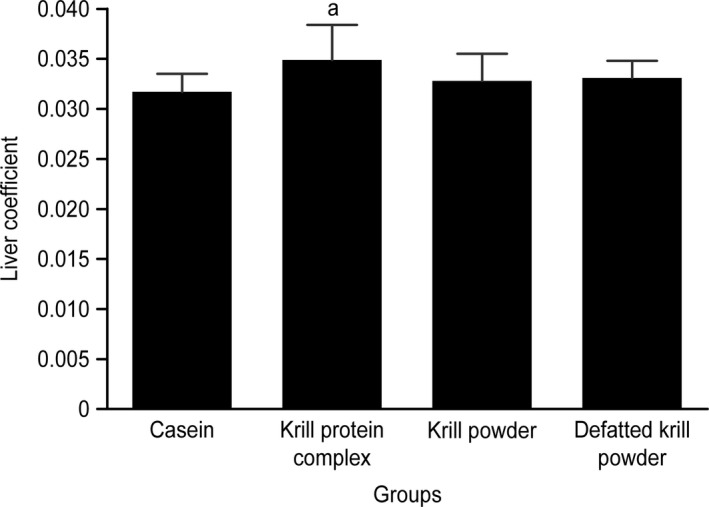
Liver coefficient in different groups. Data are presented as the mean ± *SD* (*n* = 10). ^a^ *p < *0.05, compared to casein group

The liver coefficients of the rats in the different groups are presented in Figure 2. A significant difference in the liver coefficients of rats was observed between the KPC‐ and casein‐fed groups. No significant differences in the other organ coefficients (data not shown) were observed.

We also performed a hematological test. The results shown in Table 4 demonstrate that the KPC‐fed group exhibited the highest albumin level (*p* < .05), and the Hb and transferrin levels of the KPC‐fed group were higher than those of the defatted krill powder‐fed group (*p* < .05).

**Table 4:** Hematological test

Hematological test	Casein	KPC	Krill powder	Defatted krill powder
Hb (g/L)	142.36 ± 5.43	149.78 ± 10.42	125.39 ± 8.20^ab^	121.71 ± 14.11^ab^
Albumin (g/L)	45.21 ± 2.51	51.76 ± 6.68^a^	49.02 ± 6.91^b^	45.12 ± 5.93^b^
Transferrin (ng/ml)	123.25 ± 11.88	150.32 ± 37.01	126.99 ± 17.12	77.63 ± 20.16^b^
Blood urea nitrogen (mmol/L)	6.17 ± 1.27	5.40 ± 1.05^c^	5.16 ± 0.91^c^	7.43 ± 1.67

Note

Values are presented as mean ± *SD*; significant differences are indicated with different letters in the same row (*p* < .05).^ a^ *p* < .05 compared to casein group, ^b^ *p* < .05 compared to krill protein complex group, and ^c^ *p* < .05 compared to defatted krill powder group.

Abbreviation: Hb, hemoglobin.

In contrast to the body length, the sizes of many organs, particularly the splanchnic bed, are variable, responding to lifestyle factors that influence both the energy expenditure and dietary composition and thereby regulate the energy and protein intake. The protein content of the liver, gastrointestinal tract, and kidney, among other organs, varies in response to the functional demand and might increase with increases in the dietary protein intake (Henry, Kosterlitz, & Quenouille, 1953). The liver is an organ where many functional proteins are synthesized. The current study found that the KPC favorably increased the levels of albumin and transferrin. We hypothesized that the significant difference in the liver coefficients between the KPC‐ and casein‐fed groups might be attributed to augmentation of the functional proteins and that the KPC could promote the production of functional proteins.

### 3.4 Antioxidant activity

Antioxidants are essential for life and optimal health. The link between sufficient antioxidant intake and healthy development from child to adulthood, high‐quality aging, preserved cognition, and longevity is increasingly supported by scientific findings and longitudinal clinical evaluations (Ngo et al., 2011). Antioxidant defenses comprise enzymatic and nonenzymatic compounds that decrease the steady‐state concentrations of the reactive oxygen species (ROS) and reactive nitrogen species (RNS) responsible for oxidative damage to vital biomolecules (Macedo, Bondan, & Otton, 2019).

Cellular antioxidants include low‐weight molecules, such as glutathione (GSH), carotenoids, ascorbic acid, urate, and tocopherols, as well as macromolecules, proteins, and enzymes that remove ROS and RNS, such as superoxide dismutase (SOD), GSH‐Px, and catalase (CAT) (Koutsilieri, Scheller, Tribl, & Riederer, 2002).

**Figure 3:**
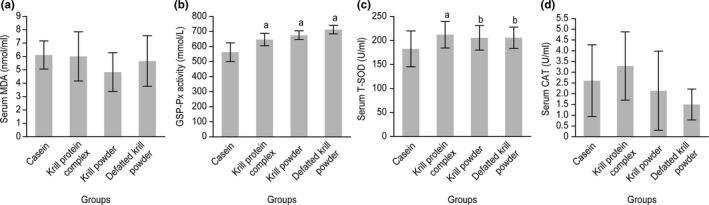
(a) Serum MDA levels (Reference); (b) serum GSH‐Px activity; (c) serum T‐SOD levels; and (d) serum CAT levels among the groups fed with casein, KPC, krill powder, and defatted krill powder. Values are presented as the mean ± *SD*; ^a^ *p* < 0.05, compared to the casein group; and ^b^ *p* < 0.05, compared to the KPC group

The results shown in Figure 3a and d indicate no significant differences in the serum levels of MDA and CAT among the casein‐fed group and the krill product‐fed groups. However, the KPC‐, krill powder‐, and defatted krill powder‐fed rats (*p* = .036, .006, and .0001, respectively) showed higher serum GSH‐Px activity than the casein‐fed rats (Figure 3b). Moreover, higher serum total superoxide dismutase (T‐SOD) activity (*p* = .029) was detected in the KPC‐fed rats compared with the casein‐fed rats (Figure 3c).

The association between antioxidant benefits and the intake of marine n‐3 PUFAs has been well researched (Higdon et al., 2000; Kiecolt‐Glaser et al., 2013; Kris‐Etherton, Harris, Appel, & Committee, 2002; Nalsen et al., 2006; Rudkowska et al., 2013). In addition, the antioxidant effects of peptides isolated from a number of aquatic species, including krill, have also been studied extensively (Kumar, Nazeer, & Jaiganesh, 2012; Nazeer, Kumar, & Ganesh, 2012; Ngo et al., 2011; Suarez‐Jimenez, Burgos‐Hernandez, & Ezquerra‐Brauer, 2012). In our study, the higher GSH‐Px activity obtained with the krill products and the higher T‐SOD activity obtained with the KPC might be due to either the existence of omega‐3 PUFAs or other antioxidant nutrients, such as zinc and selenium. A combination of nutrients, such as proteins, peptides, astaxanthin, and n‐3 phospholipids, might also independently and/or synergistically promote the biological response (Bjorndal et al., 2012). A study conducted by Ramsvik, Bjorndal, Bruheim, Bohov, and Berge (2015) showed that dietary supplementation with the phospholipid–protein complex (PPC) from krill significantly increases the total antioxidant capacity in plasma and increases the liver gene expression of mitochondrial superoxide dismutase (Sod2).

## 4 CONCLUSIONS

Our study indicates that krill products, namely the KPC, krill powder, and defatted krill powder, contain high‐quality proteins and are thus suitable dietary protein sources because their nitrogen balance and protein quality parameters are comparable to those of casein and because they can support growth. In addition, krill products increase the levels of functional proteins and are high in antioxidant nutrients. Based on their high nutritional quality and excellent health benefits, krill products appear to be a nutritious food source for human consumption, but their safety should be further studied.

## CONFLICT OF INTEREST

The authors declare that they do not have any conflict of interest.

## ETHICAL APPROVAL

All animal procedures were approved by the Animal Care and Use Committee at Ocean University of China.

## INFORMED CONSENT

Written informed consent was obtained from all study participants.

## Data Availability

The data of our study are accessible. If you would like to obtain the data, please contact us via email at cleanclearbaby@163.com.
